# Acute Psychosis as Major Clinical Presentation of Legionnaires' Disease

**DOI:** 10.1155/2016/3519396

**Published:** 2016-07-28

**Authors:** Ricardo Coentre, Amílcar Silva-dos-Santos, Miguel Cotrim Talina

**Affiliations:** ^1^Department of Psychiatry, Hospital Vila Franca de Xira, Estrada Nacional N1, 2600-009 Vila Franca de Xira, Portugal; ^2^Faculty of Medicine, University of Lisbon, Avenida Professor Egas Moniz, 1649-028 Lisboa, Portugal; ^3^CEDOC, Chronic Diseases Research Centre, Nova Medical School, Faculdade de Ciências Médicas, New University of Lisbon, Campo Mártires da Pátria, 1169-056 Lisboa, Portugal

## Abstract

We report a case of a 61-year-old woman who presented with acute psychosis as a major manifestation of Legionnaires' disease in the absence of other neuropsychiatric symptoms. Clinical history revealed dry cough and nausea. Observation showed fever and auscultation crackles in the lower lobe of the right lung. Laboratory testing demonstrated elevated C-reactive protein and lung chest radiograph showed patchy peribronchial and right lower lobe consolidation. Soon after admission, she started producing purulent sputum. Epidemiological data suggested* Legionella pneumophila *as possible cause of the clinical picture that was confirmed by urinary antigen detection and polymerase chain reaction of the sputum. She was treated with levofloxacin 750 mg/day for 10 days with complete remission of pulmonary and psychiatric symptoms. She has not had further psychotic symptoms.

## 1. Introduction

Acute psychosis as a major clinical presentation of Legionnaires' disease without other neuropsychiatric symptoms has not been reported to the best of our knowledge. Legionnaires' disease is an important disease caused by bacteria of the genus* Legionella *and is recognised as an uncommon cause of community-acquired pneumonia.* Legionella *is implicated in 0.5%–6% of community-acquired pneumonia cases in most hospital-based series [1, 2]. When not recognised or treated it has high mortality and morbidity. Respiratory symptoms caused by pneumonia are frequent but atypical pneumonia usually also includes extrapulmonary organ involvement, such as neuropsychiatric symptoms [3]. We describe a case of a 61-year-old woman with no previous significant psychiatric history who presented with acute psychosis as major initial clinical picture of Legionnaires' disease during the Vila Franca de Xira, Portugal, outbreak in 2014. The patient recovered completely after antibiotic treatment. Legionnaires' disease outbreak in Vila Franca de Xira was one of the largest community outbreaks in the world, with 375 confirmed cases and 12 (3.2%) fatalities [4]. It was identified on 7 November 2014 in Vila Franca de Xira, Portugal, a town in Lisbon's north metropolitan area, and declared controlled by 21 November 2014. Epidemiological and microbiological studies identified an industrial wet cooling system to be the probable source of infection.

## 2. Case Presentation

**Figure 1:**
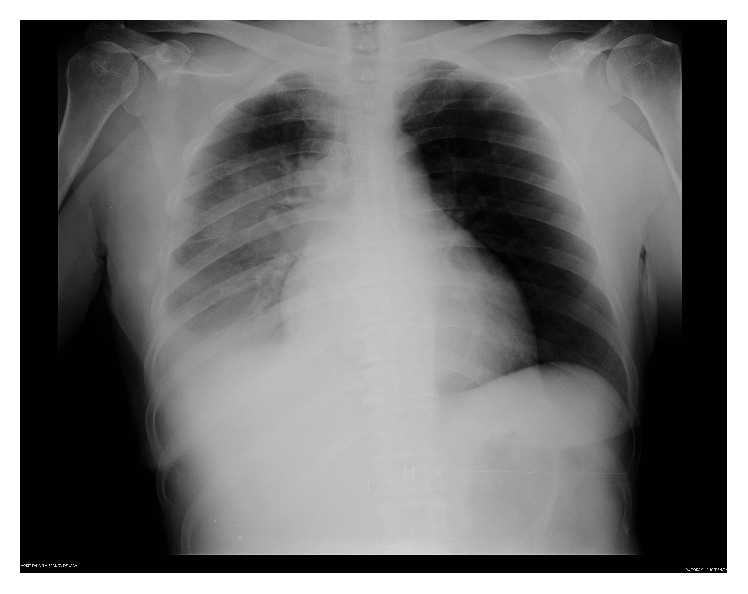
Admission chest radiograph showing patchy peribronchial and right lower lobe consolidation.

A previously healthy 61-year-old woman was admitted to the Emergency Department of Vila Franca de Xira Hospital with a 24-hour history of strange behaviour, auditory hallucinations, and systematised persecutory delusions. Her son reported that she had complained of dry cough and nausea during the preceding 5 days. On examination, she was conscious and oriented to time, place, and person but restless and suspicious. She described hearing some voices talking about her near home and she was convinced that the voices had told the hospital's nursing staff not to care for her. Her vital signs such as pulse, blood pressure, and respiratory rate were within normal limits but her body temperature was 39.6°C. Lung auscultation showed crackles in the lower lobe of the right lung. The remaining examinations including systematic review and neurological examination were normal. A few hours after admission, she produced purulent sputum. On admission, complete blood count revealed haemoglobin 10.7 g/dL, total leucocyte count 7100/*μ*L with neutrophils 79%, and platelet count 208 000/*μ*L. Liver tests demonstrated alanine transaminase 111 UI/L and aspartate transaminase 90 UI/L. C-reactive protein was 35.21 mg/dL. Kidney function and serum electrolytes were within normal limits and gasometry and routine urine tests showed no abnormalities. Chest radiograph revealed patchy peribronchial and right lower lobe consolidation (Figure 1). ECG and cranial CT were unremarkable. Because there was a* Legionella *outbreak in the area where the patient lived,* Legionella pneumophila *urinary antigen detection test was performed and was positive to* L. pneumophila serogroup 1*. During hospitalisation, results from polymerase chain reaction (PCR) of sputum detected* L. pneumophila* DNA. There was no family history of psychiatric disorders.

Nonorganic psychosis was initially considered. However, minor nonpsychiatric symptoms found during clinical history (initially only cough and nausea), epidemiological outbreak data, findings on lung auscultation, and results from blood analysis and chest radiograph rapidly put Legionnaires' disease with psychiatric symptoms as the major diagnostic hypothesis.* L. pneumophila *urinary antigen detection test and PCR of sputum confirmed* L. pneumophila* infection. The patient was treated with intravenous fluids and oral levofloxacin 750 mg/day for 10 days. Because she maintained fever in her first 2 days of hospitalisation she was also treated with oral paracetamol 1 g per dose as needed. No psychopharmacological treatment was initiated.

The patient improved dramatically within 3 days with resolution of psychosis. Clinical, radiological, and laboratory improvement of* Legionella* infection was also seen. She completed 10 days of oral levofloxacin, 7 as an inpatient and 3 after discharge. After discharge she was followed up in the pulmonology and psychiatric outpatient clinics. She resumed her employment and daily routine soon after discharge. To date, no psychotic symptoms have recurred during 18 months' psychiatric follow-up.

## 3. Discussion

This is to the best of our knowledge the first case report of acute psychosis as a major presentation of Legionnaires' disease without neurologic manifestations. There are some previous case reports about* Legionella *infection with neurological manifestations and pulmonary symptoms. Panagariya et al. reported reversible neurological syndromes with atypical pneumonia in two case reports of* L. pneumophila *infection with central nervous system (CNS) symptoms. Neurological symptoms included disorientation, headache, drowsiness, incoherence, confusion, and coma, none with psychosis [3]. Weir et al. studied 16 patients with* Legionella* infection for neurological involvement. Confusion was the most frequent neurological symptom found, but also ataxia, dysarthria, and focal signs were frequent symptoms. None of the patients presented psychotic symptoms as major extrapulmonary symptoms [5]. Other authors described case reports with psychosis attributed to other atypical bacteria. Moor and Skrinen described a 35-year-old male who developed psychosis attributable to* Mycoplasma pneumoniae *infection, who was treated with erythromycin with improvement in all symptoms [6]. Xavier et al. published a case report of a 22-year-old female with a psychotic episode probably due to* Chlamydia pneumoniae *infection. Unlike our case report, this patient developed fever only after 20 days of unsuccessful treatment with antipsychotics. She was treated with antibiotics and her psychiatric and organic symptoms improved soon afterwards [7].

It is estimated that 9%–20% of all cases of acute psychosis presenting to emergency departments are due to general medical conditions [8]. These include intracranial neoplasms and vascular diseases, drugs and toxins, infections, and vitamin deficiency (such as vitamin B12 deficiency) [9]. Atypical pneumonia, namely, caused by* C. pneumoniae*,* M. pneumoniae,* and* L. pneumophila,* frequently causes extrapulmonary manifestations such as central nervous system, hepatic or renal abnormalities, and skin or gastrointestinal involvement [10]. For example,* M. pneumoniae* infection could have extrapulmonary symptoms as many as 25% of patients infected [11]. Our patient experienced psychotic symptoms beyond subtle initial other manifestations such as dry cough and nausea. These symptoms were minor, only found during clinical history and not relevant to the patient or her family. Faced with the findings in examination and chest radiograph changes and taking into account epidemiological context (the patient lived in the area of* L. pneumophila* outbreak), the diagnostic of Legionnaires' disease was plausible. This was confirmed by urinary antigen and PCR of sputum. The patient had no significant psychiatric history and because psychotic symptoms began only after nonpsychiatric symptoms, the psychiatric diagnostic hypothesis of psychotic disorder due to a general medical condition was made (ICD-10: F06.2 organic delusional disorder [12]; DSM-5: 293.81 psychotic disorder due to Legionnaires' disease, with delusions [13]).

Legionnaires' disease is an important but relatively uncommon infection and was first recognised as a fatal cause of pneumonia when it was discovered during the investigation of a pneumonia outbreak in members of the American Legion attending their annual meeting in 1976 in Philadelphia, USA [14]. The cause of the outbreak was identified as a previously unrecognised bacterium and was designated* L. pneumophila*.* Legionella* are Gram-negative bacteria with strict growth requirements. The major natural reservoir for* Legionella* is water and it is found in natural and artificial aquatic environments. Incidence of Legionnaires' disease has been increasing in both Europe and the USA [15]. Different manifestations of the disease affect more susceptible patients as a result of immunosuppression, age older than 50 years, smoking, and chronic conditions (such as chronic lung disease) [15]. The term given to the infection was Legionnaires' disease, which refers to the pneumonic form of legionellosis. Besides age, our patient did not have any risk factors for more serious conditions. The exact mechanism by which* L. pneumophila* causes neuropsychiatric symptoms is unknown. Analogous with other infections, namely,* M. pneumoniae* infection, two major pathogenic effects have been postulated. One hypothesis includes the direct infection of the CNS by the bacteria. In previous studies,* M. pneumoniae *has been isolated from the cerebrospinal fluid using culture or PCR techniques, suggesting that organisms cross the blood brain barrier and directly invade the CNS [16]. A second hypothesis is an autoimmune mechanism with the finding of autoantibodies in the patients [17]. Unfortunately, our patient did not undergo a cerebrospinal fluid examination that may have helped explain the pathophysiology of the disease.

In our patient, treatment was directed at the underlying disease that caused the pulmonary and extrapulmonary symptoms. Therapy for Legionnaires' diseases is antibiotic treatment of the infection and management of complications and comorbidities. Several society guidelines have been published that include the treatment of Legionnaires' disease within the recommendations for community-acquired pneumonia [18, 19]. Beta-lactam antibiotics are ineffective for treatment of Legionnaires' disease.* L. pneumophila* is an intracellular pathogen residing within tissue and alveolar macrophages, and its successful treatment depends on use of antibiotics that achieve therapeutic intracellular concentrations within macrophages, such as the macrolides, fluoroquinolones, and cyclin families. Current recommended antibiotics for the treatment of Legionnaires' disease are azithromycin or levofloxacin with a minimum of 5–14 days of treatment, depending on clinical response and improvement of biomarkers. In our patient, 10 days of levofloxacin was made with apyrexy obtained after 48–72 hours' treatment and C-reactive protein reduction from 35.21 mg/dL (admission) to 3.16 mg/dL (hospital discharge).

In conclusion, our case report shows that patients with Legionnaires' disease can have extrapulmonary symptoms including acute psychosis as the major clinical picture. Acute psychosis attributed to Legionnaires' disease is reversible with appropriate antibiotic treatment.
